# Effectiveness of the Fibrinogen-Thrombin-Impregnated Collagen Patch in the Prevention of Postoperative Complications after Parotidectomy: A Single-Blinded, Randomized Controlled Study

**DOI:** 10.3390/jcm11030746

**Published:** 2022-01-29

**Authors:** Kunho Song, Chan Oh, Ho-Ryun Won, Bon Seok Koo, Da Mi Kim, Min-Kyung Yeo, Yujin Choi, Jae Won Chang

**Affiliations:** 1 Department of Otolaryngology-Head and Neck Surgery, Cancer Research Institute, College of Medicine, Chungnam National University, Daejeon 35010, Korea; songkh87@cnuh.co.kr (K.S.); ohchanny@naver.com (C.O.); hryun83@cnuh.co.kr (H.-R.W.); bskoo515@cnuh.co.kr (B.S.K.); 2 Research Institute for Medical Sciences, College of Medicine, Chungnam National University, Daejeon 35010, Korea; 3 Department of Radiology, College of Medicine, Chungnam National University, Daejeon 35015, Korea; damirad@cnuh.co.kr; 4 Department of Pathology, College of Medicine, Chungnam National University, Daejeon 35015, Korea; mkyeo83@cnuh.co.kr; 5 College of Arts and Science, Emory University, Atlanta, GA 30322, USA; yjc1999@gmail.com

**Keywords:** parotidectomy, parotid tumor, fibrinogen–thrombin–collagen sponge patch, postoperative drain, Frey’s syndrome, facial asymmetry

## Abstract

We investigated whether a fibrinogen-thrombin collagen sponge patch reduces postoperative complications of parotid gland surgery. This single-blinded, randomized controlled study included 165 patients who underwent parotid surgery for benign tumors (2018–2019) at a tertiary center. Primary outcomes were postoperative drain amount, days until drain removal, and discharge. Patients were scheduled for follow-up at 1 and 4 weeks, and 3 months after surgery. Complications including surgical site infection, pain, seroma, sialocele, salivary fistula, facial nerve palsy, Frey’s syndrome with subjective symptoms, and facial asymmetry were analyzed. After identifying confounding variables, multivariate approaches were used. Histologic analysis was performed in a mouse model of salivary gland surgery. In total, 162 patients (77, fibrinogen-thrombin collagen patch group; 85, controls) were included, with no significant between-group differences other than resected tissue. Among postoperative total drain amount and days until drain removal and discharge, the only postoperative total drain was significantly lower in the patch group than in the control group in the adjusted model. Additionally, although validation through robust trials with longer follow-up is needed, we found the potential benefit of the fibrinogen patch on Frey’s syndrome and facial asymmetry. In conclusion, fibrinogen-thrombin-impregnated collagen patches in parotidectomy can reduce postoperative drainage and improve outcomes.

## 1. Introduction

Surgery for major salivary gland tumors, including parotidectomy, is routinely performed by head and neck surgeons [1]. However, postoperative complications, such as facial palsy, wound complications, and facial asymmetry, due to tissue removal can be challenging. Facial palsy has been significantly reduced through procedural standardization and developments in surgical instrumentation [2,3,4]. Moreover, to decrease serious facial nerve complications or facial contour asymmetry, adequate or superficial parotidectomy is preferred over total parotidectomy, when possible [4].

In a previous prospective cohort study, less-extensive parotid resection was associated with a higher incidence of postoperative wound complications related to salivary leakage [5]. Early wound complications due to remnant glandular tissue, such as hematoma, seroma, sialocele, and salivary fistula, are important considerations [4]. In addition, the overall quality of life is unlikely to decline after parotidectomy if facial nerve function is preserved; most patients have delayed postoperative sequelae, including Frey syndrome, facial contour deformity at the operative site, auricular numbness, and an unsightly scar [6]. Techniques to prevent or minimize these delayed complications include fat grafts [7,8], sternocleidomastoid rotation flaps [9,10,11], temporalis muscle fascia flaps [12,13] or superficial musculoaponeurotic system (SMAS) interposition [14,15,16,17]. However, these techniques have limitations, in that they require additional incisions that can result in extra scar formation or induce donor morbidity.

Collagen patches coated with human fibrinogen and thrombin have been used extensively to improve postsurgical hemostasis [18]. A recent study covering most fields of surgery suggested that fibrinogen-thrombin-impregnated collagen patches can promote tissue sealing and reduce anastomosis failure. Such patches have been used to anastomose the gastrointestinal tract [19,20], prevent lymphoceles [21], manage air leakage after lung surgery [22], and prevent pancreatic fistula [23,24]. Thus, we postulate that a fibrinogen-thrombin-impregnated collagen patch could prevent postsurgical drainage, sialocele, or salivary fistula associated with leakage from remnant salivary tissue after parotid surgery. To the best of our knowledge, no studies have investigated the additional sealing effect of fibrinogen-thrombin-impregnated collagen patches on parotidectomy wounds.

Therefore, we prospectively investigated whether a fibrinogen-thrombin collagen sponge patch could reduce postoperative drainage by acting as a physical barrier or by applying a sealing effect to the resection area of the remnant gland to further reduce leakage-related complications. Additionally, we also investigated the effects of a fibrinogen-thrombin collagen sponge patch on Frey’s syndrome and surgical depression-related facial asymmetry, which are important delayed complications.

## 2. Materials and Methods

### 2.1. Study Design and Population

**Figure 1:**
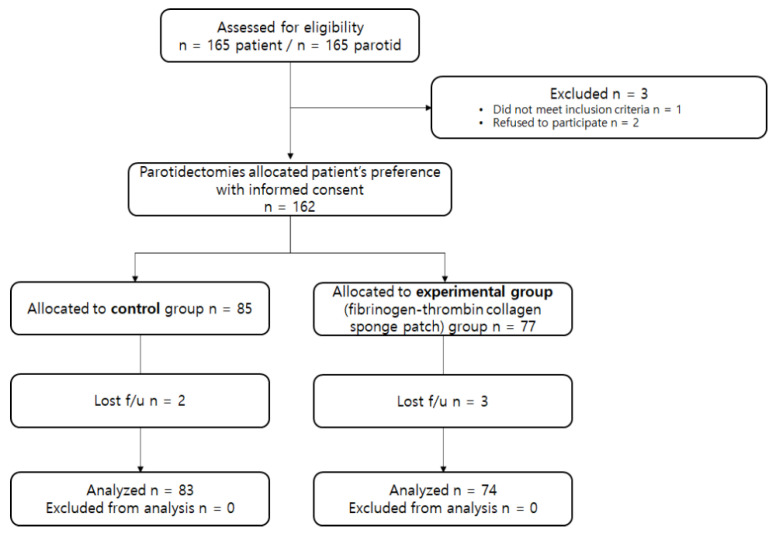
The study flow chart in line with the CONSORT statement (http://www.consort-statement.org; accessed on 5 October 2020). Numbers of participants included in this study are represented in the diagram.

This single-blinded, randomized controlled, investigator-initiated, single-center trial was approved by the Institutional Review Board of Chungnam National University Hospital (IRB No. CNUH 2018-03-067-005). Written informed consent was obtained from all patients prior to enrolment. A total of 165 patients (aged 19–70 years) who underwent parotid surgery for benign tumors diagnosed through contrast-enhanced computed tomography (CT) and ultrasonography-guided core needle biopsy from May 2018 to June 2019 in the Department of Otolaryngology-Head and Neck Surgery, Chungnam National University Hospital, were recruited. Patients with a history of head and neck radiation therapy, salivary gland surgery, chronic sialadenitis, or Sjogren’s syndrome; those under 18 years of age; and pregnant women were excluded from this study (Figure 1). Patient demographics, such as age, sex, pathologic type, and type of surgery, were recorded. Patients were assigned to the control or test groups by simple randomization using a computer-generated random number program (GraphPad QuickCalcs); in the test group, the fibrinogen-thrombin collagen sponge patch was applied at the end of parotidectomy. The randomization sequence was managed by a statistician who did not participate in the surgery and was blinded to the patients. The study adhered to the tenets of the Declaration of Helsinki and the CONSORT (CONsolidated Standards of Reporting Trials) 2010 guidelines and was registered in the Clinical Trial Registry of Korea (https://cris.nih.go.kr; registration no. KCT 0005785; principal investigator: Jae Won Chang; date of first registration: 15 January 2021).

### 2.2. Surgical Technique

**Figure 2:**
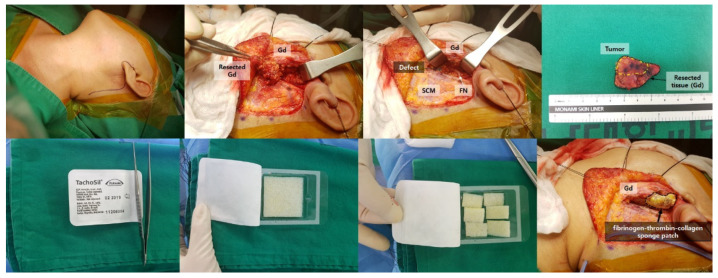
Operative procedure of the patients in the fibrinogen-thrombin-collagen sponge patch group. After superficial parotidectomy was performed using a conventional parotid surgery technique (tunnel technique), a ready-to-use fibrinogen-thrombin-collagen sponge patch (4.8 cm × 4.8 cm × 0.5 cm), was moistened in normal saline and applied to the cut surface of the parotid gland with at least 1 min of manual compression. FN, facial nerve; Gd, gland; SCM, sternocleidomastoid muscle.

All surgical procedures were standardized and performed by three different surgeons (B.S.K, H.R.W, and J.W.C). Intraoperative facial nerve monitoring was performed using the NIM-Response 3.0 (Medtronic Xomed Inc., Jacksonville, FL, USA) nerve integrity monitor. After sedation, electrodes were inserted into the orbicularis oculi and the orbicularis oris along with a ground electrode. The threshold of the monitor was set at 100 μV. After the operation, TachoSil^®^ (Takeda Pharma A/S, Roskilde, Denmark), a fixed combination of a collagen patch coated with a dry layer of human thrombin (2.0 IU) and fibrinogen (5.5 mg), was cut to suit the resection site and applied over the site with at least 3 min of gentle manual compression according to the manufacturer’s instructions. One JP drain was placed behind the incision line, and the operation was terminated after layer-by-layer repair (Figure 2). The drain was removed when the total amount of drainage per day was less than 10 mL for two consecutive days after surgery. Cefotiam (1 g bid) was prescribed postoperatively for all patients until discharge. After discharge, cefditoren pivoxil (100 mg tid) was prescribed for 1 week.

### 2.3. Data Recording and Evaluation of Surgical Outcomes

Data recorded prospectively included patient demographics, pathologic type (pleomorphic adenoma, Warthin’s tumor, and others), and type of operation (total, superficial, and partial); postoperatively, daily postoperative pain and painkiller usage, daily drain amount, the day of drain removal, facial nerve function, and the length of hospital stay were recorded. All patients were scheduled for follow-up at 1 and 4 weeks, and 3 months after surgery to counsel them on histopathology reports, wound care, and to inquire about their symptoms and complications; any complications, including SSI, seroma, salivary fistula, facial nerve palsy (transient/permanent), facial asymmetry, and Frey’s syndrome, occurring during this period were recorded. Using an online calculator (https://keisan.casio.com/exec/system/1223392149; accessed on 5 October 2020), the surface and volume of resected tissue, including tumors, were measured three-dimensionally on the assumption that the removed tissues were an elliptical sphere.

### 2.4. Definition of Specific Complications

- Surgical extent: Surgery was classified as partial parotidectomy when the facial nerve trunk was exposed but only a part of the superficial lobe was resected. There were no cases of extracapsular dissection or enucleation. When the lateral lobe of the parotid gland was totally removed, the surgery was considered a superficial parotidectomy. Total parotidectomy was defined as the complete dissection of the facial nerve and the removal of all or most of the parotid tissue lateral and medial to the nerve [25].
- Surgical site infection: SSI was defined as purulent drainage from the wound and/or microbes isolated in an aseptically obtained culture of fluid or tissue from the surgical site and/or surgeon’s diagnosis of infection based on signs or symptoms of infection (fever, pain or tenderness, localized swelling, redness, or heat) within 30 days of surgery [26].
- Seroma and sialocele: Seroma and sialocele were defined as persistent fluid collection at the surgical site lasting over 2 weeks from surgery and needing treatment [26,27].
- Salivary fistula: Salivary fistula was defined as a communication between the skin and a salivary duct or gland, through which saliva was discharged for more than 1 week [28].
- Facial nerve palsy: Postoperative facial nerve function was measured according to the House–Brackmann classification system, ranging from grade I (normal function) to grade VI (complete loss of facial motor function). On the first postoperative day, two physicians (K.S. and J.W.C.), with at least one of them being an experienced head and neck surgeon, separately performed clinical examination of all facial nerve branches. Even a minimal impairment in facial function was considered as palsy. Patients with facial weakness were scheduled for an additional follow-up at 6 months. Any facial weakness from which the patient fully recovered within 6 months after surgery was defined as temporary. When the facial palsy lasted more than 6 months, it was classified as permanent [26].
- Frey’s syndrome: Frey’s syndrome was subjectively measured by the gustatory sweat symptom of the patients during the follow-up period and an additional phone survey at the time of data analysis [26].
- Loss to follow-up: A loss to follow-up was defined as the absence of a consultation on postoperative day (POD) 30. In such a case, the patient was contacted by phone. If no answer was obtained, the patient’s family was contacted for further information about the patient’s health. If no such information was received, the patient was considered as having been lost to follow-up.

### 2.5. Facial Asymmetry

Unlike other studies that reported surgical depression using only the patient’s subjective satisfaction, we attempted to objectively analyze the surgical depression by comparing the change in facial asymmetry before and after surgery. We used two reference lines on the frontal view of all the patients enrolled in the study. A line starting from the midline glabella to the menton was defined as the median sagittal line of the facial photo. Additionally, a line starting from the upper corner of the ear to the upper center of the eyebrow was defined as the upper limit line of the face in the 2D analysis. To quantify the area, the facial photo was loaded into Photoshop 2020^®^ (Adobe, San Jose, CA, USA), and the pixel number was measured using the Marquee tool. Subsequently, the facial asymmetry was calculated by the following formula [29].

$$\frac{Pixel\;exp-Pixel\;cont\;}{Pixel\;exp+Pixel\;cont}\times 100\left(\%\right)=Asymmetry\;index\;\left(AI\right)\left(\%\right)$$

*exp*, Experimental side = operated side; *cont*, Control side = opposite side of the operated side.

$$AI_{pre}=Preoperative\;facial\;asymmetry\;indexAI_{post}=Postoperative\;facial\;asymmetry\;index\Delta AI=AI_{post}-AI_{pre}$$

$$AI_{post}=Preoperative\;facial\;asymmetry\;indexAI_{post}=Postoperative\;facial\;asymmetry\;index\Delta AI=AI_{post}-AI_{pre}$$

$$\Delta AI=AI_{post}-AI_{pre}$$

### 2.6. Three-Dimensional Photogrammetry for Facial Asymmetry Analysis

Three-dimensional (3D) facial surface data were obtained from the patients using a Vectra H2 3D scanner (Canfield Scientific, Parsippany, NJ, USA) at preoperative, immediate postoperative, and 1-month postoperative time points. The scanned data was exported in Object format (.obj) from Vectra H2 and imported to the 3Ds Max software (Autodesk, San Rafael, CA, USA). In 3Ds Max, coronal, sagittal, and transverse planes were created for the preoperative (A1) data, and the data were adjusted so that the coronal plane was in front of the earlobe, the sagittal plane was the middle of the forehead and nasal tip, and the transverse plane was the external ear canal. It was positioned as a reference. We matched the symmetry by making the parallel straight lines connecting landmarks (the lateral canthus of the eye, lateral commissure of the lip, and external auditory canal), and the median sagittal plan vertically. Using the 3-Matic Research 9.0 software (Materialise, Leuven, Belgium), the immediate postoperative (A2) and 1-month postoperative (A3) symmetrically aligned images were overlapped on the preoperative 3D image (A1) by N point registration (based on the eyes, nose, lip, and external auditory canal) and global registration. After erasing unnecessary areas (hair, neck, and back of the ears) of the three overlapping data in Zbrush (Pixologic, Los Angeles, CA, USA), we exported the data (A1), (A2), (A3) in 3Ds Max. In 3-Matic, we performed a comparison analysis for the surgical site and the corresponding normal site. The analysis results were visualized on the surgical site as a color histogram showing 12 ranges of changes of asymmetry after surgery in millimeters.

### 2.7. Statistical Analysis

The null hypothesis (H_0_) of our study is ‘fibrinogen-thrombin collagen sponge patch does not significantly reduce postoperative drainage or leakage-related complications’ and the primary endpoint is postoperative total drain amount until discharge from the hospital. Based on the results of previous studies, it was assumed that the total amount of postoperative drain in the control group would be approximately 160 mL, and TachoSil^®^ would decrease about 15%. To show a difference between the treatments at a power of 80%, in a 2-sided test, with a significance level of 5%, it was calculated that 136 patients were required (68 patients per group). Due to the clinical characteristics of the benign parotid disease, where follow-up loss is relatively common after surgery, the number of patients was established of 164 (82 patients per group). Surgical outcome analyses were conducted with the intention to treat all patients. Demographic characteristics and surgical data were analyzed with descriptive statistical methods and are presented as means with standard deviations and/or percentages. To compare the means of parametric continuous variables, a two-tailed unpaired Student’s *t*-test was used, and the chi-square or Fisher’s exact test was used to analyze the relationship between categorical variables. To identify confounder independent variables, Pearson’s correlation analysis, the t-test, one-way ANOVA, and chi-square tests were used according to the variable characteristics to measure the associations. Multivariate analysis using binary logistic regression approaches was performed to adjust confounding variables. A *p* value < 0.05 was considered significant. All statistical analyses were performed using SPSS version 23.0 (SPSS Inc., Chicago, IL, USA).

### 2.8. Data Availability

The datasets generated during and/or analyzed during the current study are available from the corresponding author on reasonable request.

## 3. Results

### 3.1. Baseline Patient Characteristics

Among 165 patients, one was excluded from the study because of having Sjogren’s syndrome and two patients did not consent to participate in the study; thus, a total of 162 patients were enrolled with informed consent and randomly assigned to the fibrinogen-thrombin-impregnated collagen patch (*n* = 77) and control groups (*n* = 85); three and two patients were lost during follow-up in each group, respectively. No major protocol violation occurred during the study. Thus, a total of 83 and 74 patients in the control group and the fibrinogen-thrombin-impregnated collagen patch group, respectively, were included in the analysis (Figure 1).

There were no significant differences in the clinical and operative characteristics, including surgeon distribution, between the two groups, except that a larger volume and wider surface of the parotid gland was resected in the patch group than in the control group (*p* = 0.033 and 0.014, respectively; Table 1).

### 3.2. Associations between Baseline Characteristics and Primary Outcome Variables

The associations between the baseline characteristics (Table 1) and outcome variables were analyzed to identify potential confounders (Table 2). Among the primary outcomes, the number of days until discharge was significantly associated with pathologic type (*p* = 0.009). No other associations were found.

### 3.3. Effect of Fibrinogen-Thrombin-Impregnated Collagen Patch on Primary Outcomes and Other Complications

**Figure 3:**
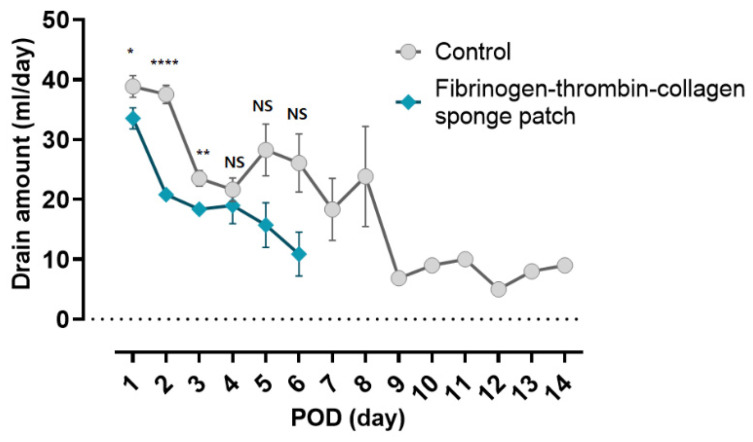
Comparison of the postoperative daily drain amount between the control group and the fibrinogen-thrombin-collagen sponge patch group. Dots represent the mean drain amount during hospitalization. Error bars represent standard errors. POD, postoperative day. NS, not significant; * *p* < 0.05; ** *p* < 0.01; **** *p* < 0.0001.

In univariate analysis of the outcome variables without adjustment, the total drain amount was reduced by 27.8% (41.0 mL) in the fibrinogen-thrombin collagen patch group compared with the control group (106.6 vs. 147.6 mL, respectively; *p* < 0.003; Table 3). Interestingly, the daily average drainage of the patch group was less than that of the control group on all postoperative days. The difference was greatest on the second day after surgery (*p* < 0.0001), suggesting a rapid decrease in the drain amount in the fibrinogen-thrombin collagen patch group (Figure 3). The number of days until drain removal was reduced by 17.7% (0.86 days) in the fibrinogen-thrombin collagen patch group compared with the control group (4.08 vs. 4.96 days, respectively; *p* < 0.003; Table 3). In addition, as shown in Figure 3, no patients had Jackson-Pratt (JP) drainage 6 days after the surgery in the fibrinogen-thrombin collagen patch group, whereas in one patient the drain was retained drain until 14 days after surgery in the control group.

No specific complications, such as seroma, hematoma, wound swelling, and pain, were observed after drain removal in both groups in the hospital. The wound condition and general postoperative health were both important in determining patients’ discharge from the hospital. Generally, if the wound was stable after drain removal, the patient was discharged 1 to 2 days after the drain removal day. Therefore, in our study, days until discharge showed a similar tendency to the drain removal days. Days until discharge were reduced by 10.6% (0.77 days) in the fibrinogen-thrombin collagen patch group compared with the control group (6.47 vs. 7.24 days, respectively; *p* < 0.031; Table 3).

After dividing the postoperative complications of parotid surgery into early and late categories, we analyzed between-group differences in their incidence. No early complications, such as surgical site infection (SSI), seroma, and salivary fistula, were observed 3 months postoperatively; thus, the results observed 1 month postoperatively were analyzed. The differences in the occurrence of early complications, such as SSI, seroma, salivary fistula, and transient facial palsy, were not statistically significant; however, complications tended to occur less frequently in the fibrinogen-thrombin-collagen sponge patch group, except for SSI (Table 3). Among the late complications, Frey’s syndrome, which is characterized by unilateral sweating and flushing of the facial skin that occurs during meals in the area of the parotid gland, was reduced by 8.1% (10.8%–2.7%) in the patch group compared with the control group (2.7% (2/74) vs. 10.8% (9/83), respectively; *p* = 0.043; Table 3). In addition, pre- and postoperative facial asymmetry 1 month after surgery, as an indicator of surgical depression caused by parotidectomy, was significantly decreased in the fibrinogen-thrombin-collagen sponge patch group than in the control group (−1.22 vs. −2.11% of ratio, respectively; *p* = 0.024; Table 3), even though the volume removed during surgery was significantly larger in the patch group than in the control group (150.36 vs. 109.64 cm^3^, respectively; *p* = 0.033; Table 1).

**Figure 4:**
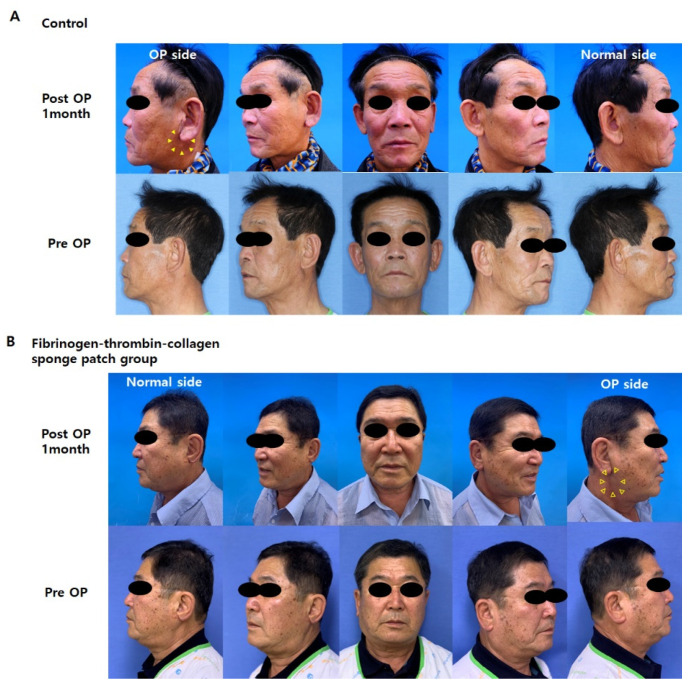
Representative facial photo images of the enrolled patients at pre- and post-operative 1 month. (**A**) Control group. A 74-year-old male underwent left superficial parotidectomy (pleomorphic adenoma, 2.6 cm × 1.9 cm × 1.9 cm). A fibrinogen-thrombin-collagen sponge patch was not applied in the surgery. The picture shows mild depression (arrowhead) on the operation site compared to normal side at 1 month after surgery. (**B**) A 55-year-old male underwent right superficial parotidectomy (pleomorphic adenoma, 3.7 cm × 2.0 cm × 1.8 cm). A fibrinogen-thrombin-collagen sponge patch (4.8 cm × 4.8 cm) was applied on the resected plane. The follow-up photo at 1 month shows good cosmetic results on the operation site (open arrowhead).

Figure 4 shows representative facial photographs before and after surgery. To quantify facial depression, facial photographs of all patients were analyzed, and the asymmetry ratio was expressed as a numerical value.

**Figure 5:**
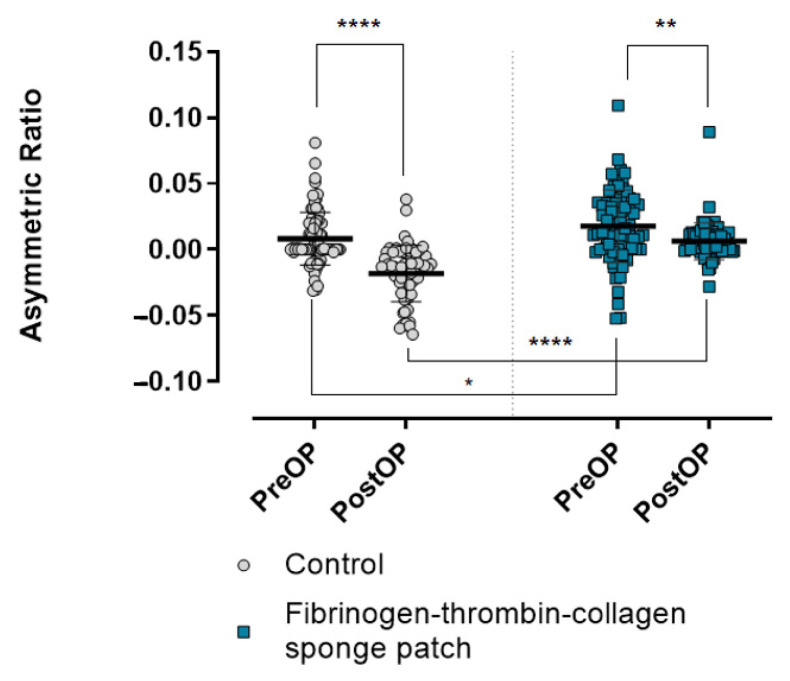
Comparison of the facial asymmetry between the control group and the fibrinogen-thrombin-collagen sponge patch group. The facial asymmetry ratio is displayed on the graph. The asymmetric ratio was calculated by the following formula: Asymmetric ratio = The ratio of the surface area of the operated side to the total face surface area-0.5. Values higher than zero mean that operative side protrudes more than the other side. The difference in the facial asymmetry ratio after the surgery was significantly less in the fibrinogen-thrombin-collagen sponge patch group than in the control group. NS, not significant; * *p* < 0.05; ** *p* < 0.01; **** *p* < 0.0001.

As shown in Figure 5, although the ratio was significantly larger in the fibrinogen-thrombin-collagen sponge patch group compared with the control group preoperatively (*p* = 0.015), the values were positive in both groups (0.0176 vs. 0.0083, respectively). Postoperatively, the facial asymmetry ratio decreased in both groups (0.0083 to −0.0181 for the control group, 0.0176 to 0.0059 for the fibrinogen-thrombin-collagen sponge patch group). Interestingly, the ratio decreased from a positive to a negative value in the control group, whereas it remained positive in the patch group. We further evaluated the effect of the patch on the facial asymmetry using 3D photogrammetry.

**Figure 6:**
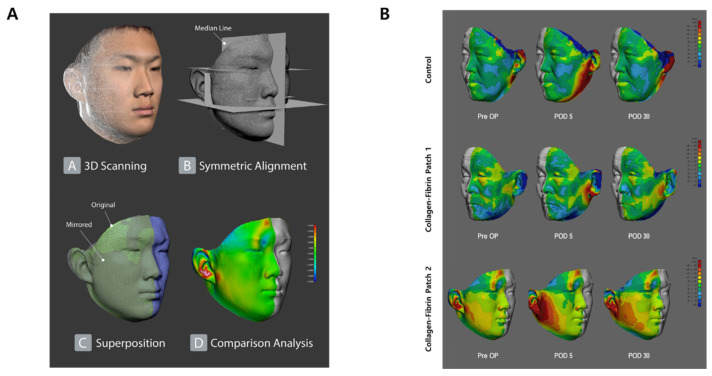
Three-dimensional (3D) surface evaluation of facial asymmetry before and after surgery. (**A**) 3D data production and processing workflow using stereophotogrammetry. (**B**) Histogram of pre- and post-operative facial asymmetry. In the control group, the difference in facial asymmetry was a negative value at 30 days postoperatively, whereas the values in fibrinogen-thrombin-collagen sponge patch group were positive in both representative patients. The color ranges visualized on the surgical site depict the range of changes of asymmetry after surgery in millimeters.

As shown in Figure 6, the parotidectomy bed was significantly edematous postoperatively (POD 5), but the preauricular area was slightly depressed in the control group, whereas it remained positive for patients in the fibrinogen-thrombin-collagen sponge patch group on POD 30.

The results of the adjusted model by regression analysis are presented in Table 4. After adjustment, the surgically resected volume was larger in the patch group than in the control group (odds ratio [OR], 1.007; 95% confidence interval [CI], 1.002 to 1.012; *p* = 0.004). The patch group had a lower total drain amount than that of the control group (OR, 0.989; 95% CI, 0.979 to 0.999; *p* = 0.038). In addition, among the complication variables, the incidence of subjective symptoms of Frey’s syndrome (mean follow-up period was 19.8 ± 10.8 months) and the severity of facial asymmetry one month postoperatively was significantly less in the patch group compared to that in the control group on multivariate analysis (*p* = 0.044 and <0.001, respectively).

### 3.4. Postoperative Evaluation of Surgical Bed Using Ultrasonography

**Figure 7:**
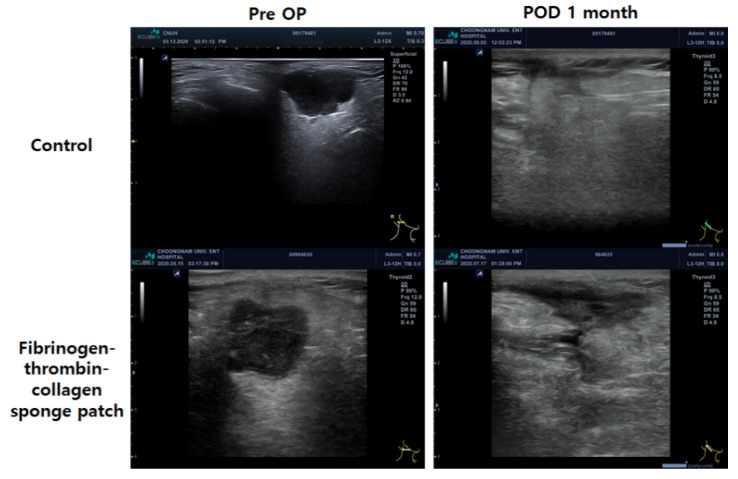
Representative preoperative and 1-month postoperative ultrasonographic images of parotid gland surgery (final diagnosis of benign parotid tumor). Preoperative transverse sonograms show well-circumscribed, multilobulated hypoechoic masses in the superficial lobe of the right parotid gland with posterior acoustic enhancement. Tumors were entirely resected through parotid surgery in both the control and fibrinogen-thrombin-collagen sponge patch groups. The 1-month postoperative transverse sonogram shows an irregularly shaped hypoechoic lesion (thickness: 0.42 cm) in the parotidectomy bed, suggestive of fibrosis. In the fibrinogen-thrombin-collagen sponge patch group, the parotidectomy defect has been replaced by markedly thicker heterogeneously hypoechoic lesions (thickness: 1.1 cm).

To confirm the presence of fibrotic neo-tissue formation in the animal experiment after fibrinogen-thrombin-collagen patch application, ultrasound examinations were performed. As shown in Figure 7, preoperative transverse sonograms revealed well-circumscribed, multilobulated hypoechoic masses in the superficial lobe of the right parotid gland with posterior acoustic enhancement. Tumors were entirely resected through parotid surgery in both control and patch groups. The 1-month postoperative transverse sonogram revealed an irregularly shaped hypoechoic lesion (thickness: 0.42 cm) in the parotidectomy bed, suggestive of fibrosis. Moreover, compared to the control group, the parotidectomy defect in the patch group had been replaced by markedly thicker heterogeneously hypoechoic lesions (thickness: 1.1 cm), which was highly suggestive of fibrotic tissue.

## 4. Discussion

This study examined the effectiveness of fibrinogen-thrombin-collagen sponge patches during parotid surgery for postoperative drainage. Our results showed that the patch helps reduce the total drainage. In the univariate analysis, the duration of drain maintenance and hospitalization were also shortened in the patch group, with a statistical significance. In accordance with the low immunogenicity of collagen, clinical experience with TachoSil^®^ [18,30] showed that the patch was non-inferior with respect to outcomes such as SSI, seroma, salivary fistula, facial nerve paralysis (transient and permanent), and postoperative pain after parotidectomy, suggesting the safety of applying the patch to the resected surface of the salivary gland where the facial nerve was exposed. In addition, most of the patients in this study were satisfied with the surgical scar at the end of the follow-up. Our data are clinically relevant because the salivary leakage that inevitably occurs due to the nature of parotidectomy was reduced. This is important because the reduction in salivary leakage is directly linked to recovery after the surgery.

The development of local wound complications, such as seroma, sialocele, and salivary fistula, which are generally considered to be due to constant fluid secretion from saliva-producing parenchyma, has been reported in 5−39% of parotid surgeries [31,32]. These complications worsen the wound and decrease the quality of life [33]. Although they inevitably occur when operating on the salivary glands, it is believed that they can be reduced by improvements in surgical techniques [26]. For instance, some surgeons suggest that techniques to reduce dead space, including drain placement, pressure dressing, or fibrin glue, can reduce the incidence of postoperative seroma [26]. Retrospective studies with approximately 100 patients and one randomized control study of 22 patients reported inconclusive results on the reduction of wound complications with the use of fibrin glue in parotidectomy [34]. To our knowledge, there is currently no study reporting the use of a fixed combination of a collagen sponge patch with a fibrinogen and thrombin layer product.

TachoSil^®^ is a ready-to-use third-generation agent based on a collagen patch coated with a mixture of human fibrinogen and thrombin [18], and was originally developed for hemostasis. However, its efficacy for tissue sealing has also been confirmed in liver, lung, and kidney surgery [34]. Its beneficial properties in terms of hemostatic efficacy and high adhesive strength have been reported for controlling diffuse oozing-type and suture-hole bleeding, and to occlude structures such as the bronchioles, lymph vessels, bile ducts, and organ ruptures [3,18]. Given these clinical properties, we hypothesized that using TachoSil^®^ as a fibrinogen-thrombin-impregnated collagen patch may reduce postoperative drainage and other complications associated with saliva leakage in parotid gland surgery. To the best of our knowledge, this is the first study evaluating the effect of a fibrinogen-thrombin-impregnated collagen patch on decreasing postoperative drainage and other complications in parotid surgery.

We also demonstrated that the application of a fibrinogen-thrombin-collagen sponge patch can prevent Frey’s syndrome and is effective for surgical depression. We were able to contact all our patients via phone to assess their subjective symptoms and found that 7% (11 of 157) reported Frey’s syndrome. Generally, 10% of patients complain of symptoms, approximately 30 to 40% admit to symptoms with questioning, and 90% have some degree of gustatory sweating with the starch iodine test [35,36]; the rate of incidence for Frey’s syndrome was low in our study [35]. As the follow-up period in this study may not have been sufficient to confirm Frey’s syndrome, its incidence may increase over time, presumably due to the time necessary for nerve regrowth. Intriguingly, Frey’s syndrome tended to occur less frequently in the fibrinogen-thrombin-impregnated collagen patch group even in the adjusted model. This is probably because the physical barrier formed by the collagen patch prevented parasympathetic nerve branches to the cutaneous sweat glands [37,38]. Numerous studies have tried to prevent the development of Frey’s syndrome with a similar concept using sternocleidomastoid muscle flaps [39], platysma muscle flaps [40], temporalis fascia flaps [41], SMAS flaps [17], adipose tissue [41], fascia lata [42], dermal matrix [37], Gore-Tex [43], and lipolyzed dura [44] with varying results. Govindaraj et al. reported that placement of allograft dermis implants decreased the incidence of Frey’s syndrome with a subjective rate of 9% vs. 3% in control vs. implanted groups, respectively, and with objective testing revealing gustatory sweating in 40% vs. 0% in the control and implanted groups, respectively [45]. Similarly, our data showed that the incidence of Frey’s syndrome decreased from 10.8% in the control group to 2.7% in the patch group. However, our data still has a limitation in that the statistical power is not high because the incidence of most complications, including Frey’s syndrome, is not high in both groups. More studies are needed to confirm the results.

In addition, because depression of the facial contour after parotidectomy remains challenging to surgeons, and facial contour asymmetry and cosmetic results may affect patients’ quality of life, surgical adjuvant techniques have been attempted to minimize surgical depression induced by resection of the gland parenchyma; these include the SMAS advancement flap, fascia lata flap, and sternocleidomastoid muscle flap [46]. However, these methods may cause donor site morbidity. To avoid this additional risk, we tried to elucidate whether the fibrinogen-thrombin collagen sponge patch could improve facial deformity. Although it is an important factor influencing the quality of life, there is no objective analysis of facial asymmetry after parotid surgery; instead, relatively subjective methods, such as questionnaires or visual analog scales, have been used [47,48]. In this study, we attempted an objective analysis using 2D and 3D photometry to compare facial contouring after parotidectomy for the first time. Initially, the asymmetry ratio was positive in both groups when the region of interest (ROI) was defined as the surgical site, due to protrusion caused by the tumor mass. However, after surgery, a significant portion of the salivary glands, including the tumor, was removed; therefore, the ratio decreased in both groups, but the degree of reduction was significantly less in the collagen patch group than that in the control group. As a result, the asymmetry index was negative in the control group, while it remained positive in the collagen patch group; we do not consider that the patch alone filled the defect. Given that, however, the number of total parotidectomies tended to be higher in the control group, and a statistically significantly larger amount of tissue was surgically removed in the fibrinogen-thrombin-collagen sponge patch group, our data highlight the possible effect of fibrinogen-thrombin-collagen sponge patch on postoperative facial asymmetry.

One previous study in a canine model histologically demonstrated that TachoSil^®^ could repair a pleural defect by providing a mechanical scaffold on which healing could proceed without negatively affecting the lung parenchyma, followed by complete biodegradation; thus, we presume that it acted as a scaffold for neo-tissue formation in our study [18]. Although the exact mechanism for fibrotic neo-tissue formation remains unknown, we demonstrated that TachoSil^®^ was replaced by fibrotic neo-tissue 1-month postoperatively, with resolved early-stage inflammation in a nude mouse salivary gland surgery model. Moreover, this was indirectly inferred on the patient’s ultrasound examination after surgery. However, in future studies, an objective comparison of soft tissue contours at least 1 to 2 years after surgery will be necessary to confirm our findings.

To investigate the sealing effect of the patch and the mechanism of relieving the depression caused by surgery, we used an animal model that mimics salivary gland surgery using ultrasonic energy, and a postoperative ultrasonographic exam for the patients of each group (Supplementary Figure S1). Our mouse model was chosen because murine and human salivary glands are similar in terms of anatomy, histology, and physiology, as previously noted by Maruyama et al. [49] Similar to the results of a previous study reporting histologic changes after using a fibrinogen-thrombin-collagen-based patch in the canine arterial bleeding model [50], we showed that TachoSil^®^ initially induces an active inflammatory reaction at the application site. We propose that it forms a physical barrier at the application site that is replaced by an organized fibrotic neo-tissue approximately 4 weeks after surgery.

This study had some major limitations. It was not double-blinded, and the surgeon was aware of which patients received the fibrinogen-thrombin-collagen sponge patch. However, it is almost impossible to blind the surgeon to this because the patch must be applied correctly during surgery using a reliable and stable technique. It is also possible that, although most of the leak emanated from the cut surface, the difference in the sealing status depending on the type (advanced bipolar energy or ultrasonic based energy) or usage method of the energy device (whether the surgeon performed it directly or the first assistant performed it, etc.); and the wound healing according to the skin flap elevation, the extent of the dissection [51], and the patient’s health status or underlying disease [2], may have accounted for part of the difference in salivary leaks and drainage amount, independent of an effect of the fibrinogen-thrombin-collagen sponge patch. There is also a possibility that surgical techniques may have differed between the three surgeons; however, all the surgeons learned the technique from the same teacher at the same institution before starting their clinical career and have performed parotidectomy by themselves for at least 5 years, suggesting that differences in skill had a minimal effect. In addition, the sample size was not large enough to evaluate complication rate, and the follow-up period was insufficient to fully evaluate the incidence of late complications. For example, Frey’s syndrome has been reported to present several years after surgery [35]. In addition, Frey’s syndrome was evaluated based on self-reporting, and not through the Minor’s test. Thus, further robust studies with larger samples sizes and longer follow-up periods may be needed to confirm the potential effect of fibrinogen-thrombin-impregnated collagen patches on Frey’s syndrome and depression of the facial contour after parotidectomy.

Despite the benefits of fibrinogen-thrombin-impregnated collagen patches shown in this study, along with the reduction in the risk of bleeding, most hemostatic agents can induce temporary inflammatory reactions and cause adhesions [52,53]. The application of a fibrin patch can significantly impede the identification and dissection of the facial nerve or remaining tissue when revision surgery is required, and appropriate care should be taken when there is a possibility of revision surgery.

## 5. Conclusions

Our data suggest that using a fibrinogen-thrombin-impregnated collagen patch may reduce salivary leakage, reduce the amount of drainage, and shorten the duration of drain maintenance and hospitalization during parotidectomy. Although robust validation through double-blind randomized controlled trials with a larger cohort is required to confirm our findings, the results of this study suggest that the application of a fibrinogen-thrombin-impregnated collagen patch could help improve postoperative outcomes of parotidectomy.

## Figures and Tables

**Table 1:** Baseline patient characteristics.

	Control Group (*n* = 83)	Collagen-Fibrin Patch Group (*n* = 74)	*p* Value
Age (year)	53.00 ± 15.53	55.55 ± 13.94	0.282 ^§^
Sex (n, %)			0.256 ^†^
Male	49 (59.0%)	37 (50.0%)	
Female	34 (41.0%)	37 (50.0%)	
Diabetes mellitus			
Yes	16 (19.3%)	8 (10.8%)	0.141 ^†^
No	67 (80.7%)	66 (89.2%)	
Side			0.467
Right	35 (42.2%)	27 (36.5%)	
Left	48 (57.8%)	47 (63.5%)	
Pathologic type (n, %)			0.669 ^†^
Pleomorphic adenoma	50 (60.2%)	45 (60.8%)	
Warthin’s tumor	28 (33.7%)	22 (29.7%)	
Others	5 (6.0%)	7 (9.5%)	
Treatment methods			0.074 ^†^
Total parotidectomy	1 (1.2%)	6 (8.1%)	
Superficial parotidectomy	68 (81.9%)	60 (81.1%)	
Partial parotidectomy	14 (16.9%)	8 (10.8%)	
Resected tissue volume (cm^3^)			
Parenchyma	109.64 ± 96.67	150.36 ± 121.87	0.033 ^§^*
Tumor	65.70 ± 76.30	80.11 ± 117.88	0.425 ^§^
Resected tissue surface area (cm^2^)			
Parenchyma	109.11 ± 65.07	139.00 ± 74.35	0.014 ^§^*
Tumor	73.09 ± 56.63	77.63 ± 73.68	0.707

Data presented as *n* (%) or mean ± SD; *: statistically significant, ^§^: Student’s *T* test, ^†^: χ^2^ test.

**Table 2:** Associations between baseline characteristics and primary outcome variables.

		Drain Amount (mL)		Days until Drain Removal (Days)		Days until Discharge (Days)	
		Mean ± SD	*p* Value; *r* Value	Mean ± SD	*p* Value; *r* Value	Mean ± SD	*p* Value; *r* Value
Age	54.20 ± 14.81	128.28 ± 90.73	0.953 ^¥^; 0.0.001	4.54 ± 1.89	0.955 ^¥^; 0.0001	6.88 ± 2.27	0.483 ^¥^; 0.0003
Sex	Male (*n* = 86)	134.68 ± 98.98	0.332 ^§^	4.50 ± 1.97	0.759 ^§^	6.97 ± 2.34	0.603 ^§^
	Female (*n* = 71)	120.52 ± 79.64		4.59 ± 1.79		6.77 ± 2.20	
Diabetes Mellitus	Yes	114.90 ± 57.65	0.434 ^§^	3.92 ± 1.69	0.078 ^§†^	6.50 ± 2.73	0.376 ^§^
	No	130.69 ± 95.46		4.66 ± 1.90		6.95 ± 2.18	
Side	Right (*n* = 62)	132.91 ± 101.67	0.607 ^§^	4.41 ± 1.83	0.485 ^§^	6.74 ± 1.87	0.543 ^§^
	Left (*n* = 95)	125.25 ± 83.26		4.63 ± 1.93		6.97 ± 2.50	
Pathologic type	Pleomorphic adenoma (*n* = 95)	135.36 ± 101.83	0.136 ^⁋^	4.74 ± 1.94	0.061 ^⁋^	7.01 ± 2.51	0.026 ^⁋^*
	Warthin’s tumor (*n* = 50)	108.46 ± 66.36		4.04 ± 1.55		6.32 ± 1.23	
	Other benign tumors (*n* = 12)	154.74 ± 74.57		5.08 ± 2.43		8.17 ± 3.01	
Treatment methods	Total parotidectomy (*n* = 7)	126.23 ± 91.64	0.480 ^⁋^	4.56 ± 1.97	0.885 ^⁋^	6.95 ± 2.42	0.598 ^⁋^
	Superficial parotidectomy (*n* = 128)	147.40 ± 92.22		4.54 ± 1.44		6.68 ± 1.43	
	Partial parotidectomy (*n* = 22)	105.57 ± 67.36		4.54 ± 1.89		6.14 ± 1.35	
Resected tissue volume (cm^2^)	Parenchyma	128.28 ± 90.73	0.545 ^¥^; 0.0.003	4.54 ± 1.89	0.432 ^¥^; 0.005	6.88 ± 2.27	0.391 ^¥^; 0.005
	Tumor		0.768 ^¥^; 0.0.001		0.916 ^¥^; 0.000		0.978 ^¥^; 0.000
Resected tissue surface area (cm^2^)	Parenchyma	128.28 ± 90.73	0.513 ^¥^; 0.0.006	4.54 ± 1.89	0.333 ^¥^; 0.007	6.88 ± 2.27	0.360 ^¥^; 0.006
	Tumor		0.745 ^¥^; 0.0.001		0.893 ^¥^; 0.000		0.979 ^¥^; 0.000

^§^: Student’s T test, *: statistically significant, ^⁋^: One-way ANOVA, ^¥^: Linear regression test.

**Table 3:** The result of primary outcome and complications between groups.

	Control Group (*n* = 83)	Collagen-Fibrin Patch Group (*n* = 74)	*p* Value
Total drain amount (ml)	147.60 ± 107.95	106.60 ± 60.04	0.003 ^§^*
Days until drain removal (POD)	4.96 ± 2.06	4.08 ± 1.57	0.003 ^§^*
Days until discharge (POD)	7.24 ± 2.58	6.47 ± 1.79	0.031 ^§^*
Early complication			
SSI	1 (1.2%)	2 (2.7%)	0.457 ^††^
Seroma	24 (28.9%)	16 (21.6%)	0.295 ^†^
Salivary fistula	7 (8.4%)	1 (1.4%)	0.045 ^††^
Facial nerve palsy (transient)	10 (12.0%)	5 (6.8%)	0.197 ^†^
Late complication			
Facial asymmetry	−2.13	−1.17	0.015 ^†^*
Frey’s syndrome	9 (10.8%)	2 (2.7%)	0.043 ^††^*
Facial nerve palsy (permanent)	1 (1.2%)	0 (0.0%)	0.529 ^††^
Postoperative pain (VAS)	1.42 ± 1.43	1.34 ± 1.24	0.127 ^§^
Painkiller usage (/day)	0.31 ± 0.64	0.27 ± 0.65	0.615 ^§^

SSI, Surgical site infection; POD, Post-operative day; Data presented as *n* (%) or mean ± SD; ^§^: Student’s T test; *: statistically significant, ^†^: χ^2^ test, ^††^: Fisher’s exact test.

**Table 4:** Multivariate logistic regression model for drain amount and day until drain removal.

Variables	B	S.E.	OR	95% CI	*p* Value
Pathologic type					
Pleomorphic adenoma					0.066
Warthin’s tumor	−0.085	0.487	0.434	(0.167~1.127)	0.086
Others	1.074	0.793	2.926	(0.618~13.858)	0.176
Tissue volume (cm^3^)	0.007	0.002	1.007	(1.002~1.012)	0.004 **
Total drain (mean, mL)	−0.011	0.005	0.989	(0.979~0.999)	0.038 *
Days until drain removal (days)	0.213	0.226	1.238	(0.795~1.927)	0.345
Days until discharge (days)	−0.131	0.144	0.878	(0.661~1.164)	0.365
Frey syndrome	−1.729	0.857	0.177	(0.033~0.953)	0.044 *
Facial asymmetry	0.335	0.960	3.527 × 10^14^	(2506076.788~4.964 × 10^22^)	<0.001 ***
−2*LL* = 148.940, NagelKerke R^2^ = 0.344, Hosmer & Lemeshow test: χ^2^ = 14.465 (*p* = 0.070)					

S.E., standard error; OR, odds ratio; CI, confidence interval. *: <0.05; **: <0.01; ***: <0.001, statistically significant.

## Data Availability

The data presented in this study are available on request from the corresponding author. The data are not publicly available due to privacy or research ethics.

## Supplementary Materials

The following are available online at https://www.mdpi.com/article/10.3390/jcm11030746/s1, Figure S1: In vivo post-operative evaluation of collagen-fibrin patch in the murine salivary gland surgery.
