# Supplementary material for: Effectiveness of the Fibrinogen-Thrombin-Impregnated Collagen Patch in the Prevention of Postoperative Complications after Parotidectomy: A Single-Blinded, Randomized Controlled Study
Source: J Clin Med. 2022 Jan 29;11(3):746. doi: 10.3390/jcm11030746 (PMC8836986; doi:10.3390/jcm11030746)
Supplement: Supplementary file 1 [file jcm-11-00746-s001.zip › jcm-1579756-supplementary.pdf]

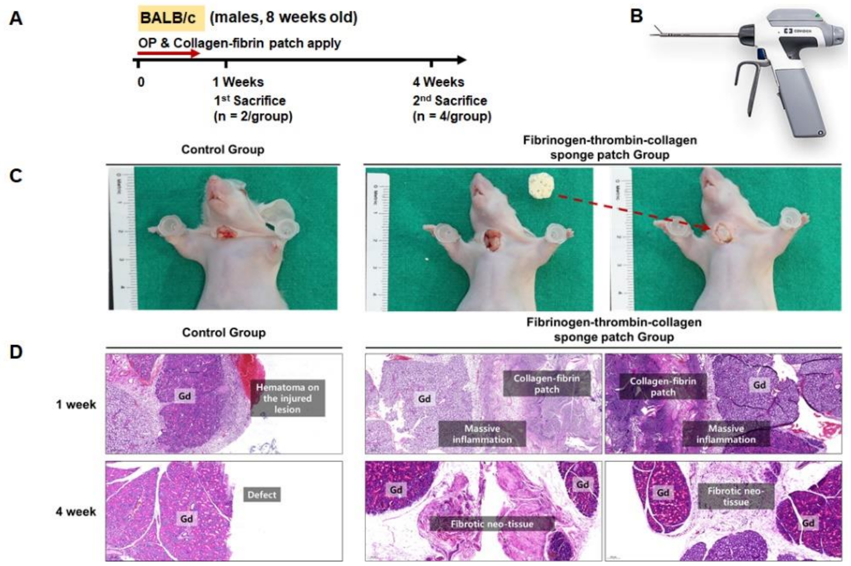

Figure S1. *In vivo* post-operative evaluation of collagen-fibrin patch in the murine salivary gland surgery.

Twelve male BALB/c nude mice were randomly divided into two groups of six animals each: control group, no care was provided for the salivary defect. In the fibrinogen-thrombin-collagen sponge patch group, the salivary defect was treated with an absorbable fibrinogen-thrombin-collagen sponge patch (1 × 1 cm^2). Two mice per group were euthanized for early histological examination 1 week after surgery, and the salivary glands of the remaining mice were examined 4 weeks after surgery. (**A**) Schema of the experimental schedule. (**B**) Energy device used in the experiment. (**C**) Surgical procedure for control and treatment group. (**D**) Histologic examination with H&E staining. In the fibrinogen-thrombin-collagen sponge patch group, irregularly shaped, light-pink-stained neo-tissue filled the salivary gland defect, suggesting fibrosis. Gd, gland; H&E, hematoxylin and eosin.

Experimental animals

After a 1-week acclimatization period, 12 male BALB/c nude mice (Orient Bio, Seongnam, Korea) were anesthetized by isoflurane and sevoflurane (Baxter, Deerfield, Illinois, USA) inhalation. After disinfection with 10% povidone iodine and 70% alcohol, we made an incision with a no. 15 scalpel and scissors at the neck midline and then made a 1×1-cm^2 sized uniform defect on the salivary gland using a cordless ultrasonic dissector (Sonicision™, Covidien, Mansfield, MA, USA). The mice were randomly divided into two groups of six animals each: in the control group, no care was provided for the salivary defect; in the fibrin-collagen patch group, the salivary defect was treated with an absorbable fibrin-collagen patch (1×1 cm^2). All mice were maintained at a pathogen-free facility, and the experimental procedures were conducted under institutional guidelines that comply with national laws. The study protocols were approved and performed in accordance with the guidelines of the Institutional Animal Care and Use Committee of Chungnam National University. To investigate the early response of the surgical wound and fibrinogen-thrombin collagen sponge patch, two mice were euthanized 1 week after the treatment. The histologic examination was performed 4 weeks after the treatment under light

microscopy. For histopathology, the salivary glands of the mice were removed carefully, fixed overnight in 4% paraformaldehyde, and embedded in paraffin using routine procedures. For each organ, three different areas were analyzed histologically. Paraffin sections (5 μm) were stained with H&E.
